# International Brazilian Journal of Urology reached the biggest Impact Factor of its history - 1,541

**DOI:** 10.1590/S1677-5538.IBJU.2021.05.01

**Published:** 2021-07-23

**Affiliations:** 1 Universidade do Estado do Rio de Janeiro Unidade de Pesquisa Urogenital Rio de JaneiroRJ Brasil Unidade de Pesquisa Urogenital - Universidade do Estado do Rio de Janeiro - Uerj, Rio de Janeiro, RJ, Brasil.; 2 Hospital Federal da Lagoa Rio de JaneiroRJ Brasil Serviço de Urologia, Hospital Federal da Lagoa, Rio de Janeiro, RJ, Brasil.

This is a historical number for our Journal. We are pleased to announce that International Brazilian Journal of Urology reached the biggest impact factor of its history - **1,541**. The editorial board and the Brazilian Urology Society are very proud with consolidation of the International Brazilian Journal of Urology as one of the most relevant in the dissemination of urology research worldwide.

The September-October 2021 number of *Int Braz J Urol*, the 13th under my supervision, presents original contributions with a lot of interesting papers in different fields: Renal Cell Carcinoma, Bladder Cancer, SARS-CoV-2 and Urology, Basic Research applied to prostatic diseases, Premature ejaculation, Reconstructive urology, Lower urinary stones, Ureteral Stones, Lower urinary tract symptoms in children and Xanthogranulomatous Pyelonephritis. The papers came from many different countries such as Brazil, USA, Iran, Israel, Colombia and Singapure, and as usual the editor's comment highlights some of them.

Dr. Sharma and colleagues from India performed in page 921 (1) a nice sistematic review about the on-demand use of tramadol in premature ejaculation (PE) and concluded that Tramadol appears to be an effective drug for the management of PE with a low propensity for serious adverse events. However, evidence obtained from authors study is of low to moderate quality. Furthermore, effective dose and duration of therapy remain elusive.

Dr. Dispagna and colleagues from USA (2) present in page 935 an important narrative review about the Management of Variant Histology in Renal Cell Carcinoma (RCC) and concluded that clinical management should be considered and adjusted for patients with non- clear-cell RCC histological variants based on tumor subtype and genetic alterations.

Dr. Zekan and colleagues from Department of Genitourinary Oncology, H. Lee Moffitt Cancer Center & Research Institute, Tampa, FL, USA under supervision of Dr. Philippe Spiess (3) present in page 943 an important systematic review about the Prognostic predictors of lymph node metastasis in Squamous cell carcinoma (SCC) of the penis and concluded that a multitude of factors are associated with metastasis of SCC of the penis to inguinal lymph nodes, which is associated with poor clinical outcomes. The above factors, most strongly lymphovascular invasion, grade, and node positivity, may be considered when constructing a nomogram to risk-stratify patients and determine eligibility for prophylactic inguinal lymphadenectomy.

Dr. Alam and colleagues from, USA (4) present in page 957 a nice narrative review about considerations in the management and treatment of lower pole stones and concluded that lower pole stones can pose amplified anatomical considerations that influence surgical success beyond stone size alone. The selected treatment approach should account for attendant risks and benefits of the intervention within the context of patient preferences and outcome expectations.

Dr. Vasconcelos and colleagues from Brazil (5) present in page 969 a nice study about the association between attention-deficit/hyperactivity and lower urinary tract symptoms in children and adolescents in a community setting and concluded that Children and adolescents, recruited in a general pediatric outpatient clinic, with symptoms of attention-deficit/hyperactivity disorder (ADHD) symptoms are 2.3 times more likely to have LUTS. The combined type of ADHD was the most commonly associated with LUTS. Urgency and holding maneuvers were most prevalent symptoms in children and adolescents with ADHD symptoms. These findings support that all children with ADHD should be addressed for LUTS and vice versa. In page 979 Prof. Andrew Coombs (6) in a very nice editorial comment show some aspects of this important topic.

Dr. Falahatkar and colleagues from Iran (7) present in page 982 a randomized double-blind clinical trial about Efficacy of tamsulosin versus tadalafil as medical expulsive therapy on stone expulsion in patients with distal ureteral stones and concluded that Tamsulosin as medical expulsive therapy is more effective for distal ureteric stones with less need for analgesics and less stone expulsion time than tadalafil.

Dr. Frumer and colleagues from Israel (8) present in page 997 an interesting study about Sars-Cov 2 and Urological Emergencies and concluded that the general lockdown was accompanied by a significant decrease in common urological presentations to the emergency room. This change occurred across the clinical severity spectrum of renal colic, hematuria, and urinary retention. In the short term, it appears that patients who sought treatment did not suffer from complications that could be attributed to late arrival or delay in treatment. The long-term implications of abstinence from seeking emergent care are not known and require further investigation.

Dr. Anaissie and colleagues from, USA (9) present in page 1006 an important study about the role of radical cystectomy (RC) and urinary diversion (UD) in post-operative complications and concluded that RC+UD, as compared to UD alone, is associated with an increased risk of major complications, including bleeding needing transfusion and venous thromboembolism. Additionally, continent UD had a higher risk of post-operative complication than ileal conduit.

Dr. Oliveira and colleagues from Brazil (10) present in page 1020 an interesting translational study about the prostatic alterations associated to early weaning and its relation with cocoa powder supplementation in adult wistar rats and concluded that early weaning resulted in hyperglycemia and important morphological changes in the prostate. In contrast, dietary supplementation with cocoa powder attenuated these effects on the metabolism and prostatic histoarchitecture, proving to be a good nutritional treatment strategy.

Dr. Favorito and colleagues from Brazil (11) present in page 1032 an important study about a new option to prevent fistulas in anterior urethroplasty in patients with kippered urethra: the tunica vaginalis flap (TVP) and concluded that a urethroplasty with TVF technique may be a viable method for repairing penile urethral erosions, but further studies are required with a bigger sample to confirm the results.

We hope that readers will enjoy the present number of the International Brazilian Journal of Urology in this very difficult times of COVID-19.
